# Severe asthma exacerbation: role of acute *Chlamydophila pneumoniae *and *Mycoplasma pneumoniae *infection

**DOI:** 10.1186/1465-9921-9-48

**Published:** 2008-05-30

**Authors:** Roberto Cosentini, Paolo Tarsia, Ciro Canetta, Giovanna Graziadei, Anna Maria Brambilla, Stefano Aliberti, Maria Pappalettera, Francesca Tantardini, Francesco Blasi

**Affiliations:** 1 Emergency Medicine Department, Fondazione IRCCS Ospedale Maggiore Policlinico, Mangiagalli e Regina Elena, Gruppo NIV Policlinico, Milan, Italy; 2 Institute of Respiratory Diseases, University of Milan, Fondazione IRCCS Ospedale Maggiore Policlinico, Mangiagalli e Regina Elena, Gruppo NIV Policlinico, Milan, Italy

## Abstract

**Background:**

Chlamydophila pneumoniae and Mycoplasma pneumoniae are associated with acute exacerbation of bronchial asthma (AEBA). The aim of this study was to evaluate the correlation between these acute bacterial infections and the severity of AEBA.

**Methods:**

We prospectively analysed consecutive patients admitted to the Emergency Department with acute asthma exacerbation. In every patient peak expiratory flow (PEF) measurement was performed on admission, and spirometry during follow-up. Serology for Chlamydophila and Mycoplasma pneumoniae was performed on admission and after 4–8 weeks.

**Results:**

Fifty-eight patients completed the study. Acute atypical infections (AAI) was observed in 22/58 cases; we found single acute C. pneumoniae in 19 cases, single acute M. pneumoniae in 2 cases, and double acute infection in one case. Functional impairment on admission was greater in patients with AAI than in patients without AAI (PEF 205 ± 104 L/min vs 276 ± 117 p = 0.02) and persisted until visit 2 (FEV1% 76.30 ± 24.54 vs FEV1% 92.91 ± 13.89, p = 0.002). Moreover, the proportion of patients who presented with severe AEBA was significantly greater in the group with AAI than in the group without AAI (15/22 vs 12/36, p = 0.01; OR 4.29, 95% CI 1.38–13.32).

**Conclusion:**

Our data suggest an association between acute atypical infection and a more severe AEBA.

## Background

Acute exacerbations of bronchial asthma (AEBA) represent an important healthcare problem, accounting for a high rate of morbidity and mortality. Furthermore, increased disease burden and asthma symptoms frequently persist for at least 1 month after emergency department discharge following an asthma exacerbation [1].

The role of respiratory infections in asthma is well known; the micro-organisms most commonly involved are viruses and atypical bacteria, such as *Mycoplasma pneumoniae *and *Chlamydophila pneumoniae *[2,3].

*C. pneumoniae *and *M. pneumoniae *represent an important cause of human respiratory tract diseases. These agents are involved in upper respiratory tract infections acute bronchitis and exacerbations of chronic bronchitis, and pneumonia [4-6]. *C. pneumoniae *infection has been implicated in severe chronic asthma [7-9], whereas other groups demonstrated that *C. pneumoniae *and *M. pneumoniae *causes AEBA both in children and adults [3,10,11]. A recent review indicates that the high proportion of these studies that have reported a link between AEBA and *C. pneumoniae *and/or *M. pneumoniae *infection suggests that these pathogens may play a significant role in such exacerbations [12].

However, to date the relationship between acute atypical infection (AAI) and the degree of severity of acute asthma exacerbation has not been yet evaluated. Should this association be observed, then antibiotic treatment against atypical bacteria could play a role in the management of acute asthma attack.

Therefore, the aim of this study is to evaluate a possible correlation between acute *Mycoplasma *and *Chlamydia pneumoniae *infection and the severity of manifestation of acute asthma exacerbation.

## Methods

This was an observational prospective study of consecutive patients with acute exacerbation of asthma admitted to the Emergency Room of Fondazione IRCCS Ospedale Maggiore Policlinico, Mangiagalli e Regina Elena Hospital, Milan, Italy, between January 2004 and December 2004. Acute exacerbation of bronchial asthma (AEBA) was defined as following: episodes of rapidly progressive increase in shortness of breath, cough, wheezing, or chest tightness, or some combination of these symptoms necessitating a non-scheduled visit, and associated to a decrease of respiratory airflow quantified by measurements of peak expiratory flow (PEF) or FEV1 [13]. Severe exacerbation of asthma was defined when PEF on admission was <50% according to BTS criteria [14]. We chose the PEF value measurement as the only criterion for acute severe asthma exacerbation in Emergcency Room since it is a direct and reproducible measurement easily controlled in the follow-up.

Subjects satisfying criteria for acute exacerbation of asthma were eligible for the study if all the following inclusion criteria were fulfilled: 1) age > 18 yrs; 2) history of typical bronchial asthma > 6 mo and 3) smoking history < 10 pack/yrs.

Patients with a radiological diagnosis of pneumonia or with impaired consciousness on admission were excluded from the study.

On admission, past medical history and active medications were collected; every patient underwent the following examinations: PEF measurements (best out of three), spirometry, an oropharyngeal swab specimens and a blood sample for serologic testing.

Patients were treated according to British Thoracic Society guidelines, considering the degree of severity [14]. In particular, patients presenting with a severe acute attack were treated with systemic steroids, whereas those with a non-severe attack were treated with topic steroids.

Follow-up was performed at 2–4 days (Visit 1), at 10–14 days (Visit 2), and at 4–8 weeks (Visit 3), including functional evaluation by spirometry. A second blood sample was drawn for serologic testing (convalescence phase) 4–8 weeks after the admission.

Serologic tests included IgG and IgM for *C. pneumoniae *(microimmunofluorescence test, Labsystems, Helsinki, Finland), and IgG and IgM for *M. pneumoniae *(ELISA test, Pantec, Turin, Italy). On oropharyngeal swab specimens a nested-polymerase chain reaction (PCR) technique for the detection of *C. pneumoniae *DNA [15] was performed.

Acute *C. pneumoniae *infection was defined as IgM titre ≥ 1:16 or a fourfold increase in the specific IgG [16]. Acute *M. pneumoniae *infection was defined as IgM titre ≥ 1:16 or a fourfold increase in the specific IgG [17]. The person performing the MIF test was blinded to the patient diagnosis and characteristics.

Patients were divided into two groups according to the presence or the absence of acute *Chlamydia *and/or *Mycoplasma pneumoniae *infection (AAI). The two groups were compared according to the degree of functional impairment.

A sub-analysis was performed among the group of patients with acute atypical infection in order to evaluate the degree of functional impairment between the two different pathogens.

Local IRB review was performed and informed consent was obtained from all patients on admission to the study.

### Statistical analysis

All data were statistically analyzed with SPSS version 10.1 (Chicago, Il). A descriptive statistics at baseline with continuous data expressed as a mean or median (depending on distribution) ± SD was performed, and data were compared between the group with AAI and the group without AAI using ANOVA test. Baseline categorical data between the two groups were compared by the χ^2 ^test or Fisher's exact test when appropriate. p value < 0.05 was considered statistically significant. Among the group with AAI, the same test was applied to compare continuous data between the group with acute *C. pneumoniae *infection and the group with acute *M. pneumoniae *infection.

## Results

**Figure 1:**
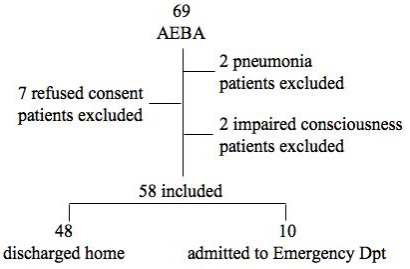
Flowchart of the study.

Sixty-nine patients were admitted to our Emergency Room and screened for the protocol. Two patients were excluded for impaired consciousness, 2 were excluded for radiological diagnosis of pneumonia, and 7 refused consent. Fifty-eight patients have been enrolled. Ten patients have been admitted to hospital, and 48 have been discharged. All 58 patients completed the follow-up (Figure 1).

Thirty-four (59%) were males, mean age was 38.1 ± 11.8 yrs, 2 were current smokers, 30 (52%) had a history of atopy, 11 (19%) were on inhaled corticosteroids before exacerbation, and 19 (33%) had a recent history of acute upper respiratory infection. Mean PEF on admission was 253.8 ± 121.1 (58 pts) and mean FEV1 (30 pts) 1.39 ± 0.63; spirometry was not performed in all patients because, in keeping with patient's best interest, treatment was not delayed and in some cases was initiated before spirometry.

Acute serologically demonstrated atypical infection was found in 22/58 patients (38%). Single acute *C. pneumonia *infection was observed in 19 out of 22 patients (86%), with a four-fold increase in IgG titre after 4–8 weeks; no IgM titre ≥ 1:16 was found.

Serologic evidence of single acute *M. pneumoniae *infection was found in only 2 out of 22 patients (9%) with a positive IgM titre. In one case, the acute *M. pneumoniae *infection was associated with acute *C. pneumoniae *infection (Table 1). PCR for *C. pneumoniae *DNA oropharyngeal swab was positive in 13/20 (sensitivity = 65%), and in 1/38 cases without acute *C. pneumoniae *infection (specificity = 93.7%).

**Table 1:** Serologically demonstrated acute atypical infection (AAI) in the study population (22/58 patients)

**Pts with AAI**	**22**	
*C. pneumoniae*	19	GMT IgG on admission 38.4 GMT IgG after 4–8 weeks 442.48
*M. pneumoniae*	2	IgM positive
*M. pneumoniae *and *C. pneumoniae*	1	IgM positive IgG on admission 1:64 IgG after 4–8 weeks 1:512

Demographic and history characteristics distribution between the two groups is shown in Table 2. On admission, PEF values in the 22 subjects with serological AAI were significantly lower when compared with measurements in the 36 patients without AAI (205 ± 104 vs 277 ± 117, p = 0.023). In addition, mean predicted PEF values according to sex, age, and height was comparable between subjects with and without AAI (546 ± 85 vs 510 ± 102, p = 0.159). However, the mean PEF percentage values of the predicted values were significantly lower in subjects with acute atypical infection (38.3 ± 18.3 vs 55.3 ± 19,5, p = 0.002).

**Table 2:** Demographic and history characteristics of the study population according to acute atypical infection (AAI)

	patients with AAI (22)	patients without AAI (38)	**p**
Males	13	18	0.501
Age (yrs)	39.3 ± 13.7	37.4 ± 10.7	0.546
Current smokers	1	1	0.631
Atopy	12	18	0.737
Inhaled steroids (before exacerbation)	5	6	0.568
Clinical symptoms of acute upper airway infection	7	12	0.905

In the subgroup of thirty patients where spirometry was performed on admission, FEV1% predicted values in the 11 patients with AAI were significantly lower than in the other 19 subjects without AAI (39.73 ± 19.64 vs 58.53 ± 20,43, p = 0.02).

Considering the severity of acute attacks according to BTS criteria [14] (severe = PEF <50%, 27 patients; non-severe, PEF > 50%, 31 patients); the proportion of patients with acute severe attack was significantly greater in the group with AAI than in the group without AAI (15/22 vs 12/36, p = 0.010; OR 4.29, 95% CI 1.38–13.32) (Table 3).

**Table 3:** Lung function in the two different groups of patients with acute atypical infection (AAI) and patients without AAI during the study period.

	**Patients with AAI (22)**	**Patients without AAI (36)**	p
PEF predicted (L/min)	546.9 ± 85.4	509.8 ± 101.9	0.159
PEF admission (L/min)	205.9 ± 104.1	276.9 ± 117.3	0.023
PEF % of predicted on admission	38.3 ± 18.3	55.3 ± 19.5	0.002
FEV1% on admission	39.73 ± 19.64 (11 pts)	58.53 ± 20.43 (19 pts)	0.02
FEV1% Visit 1 (2–4 days)	70.91 ± 25.6	89.14 ± 17.07	0.002
FEV1% Visit 2 (10–14 days)	76.30 ± 24.54	92.91 ± 13.89	0.002
FEV1% Visit 3 (4–8 weeks)	85.05 ± 19.13	92.26 ± 14.44	0.114
Acute severe attack (number of patients)	15	12	0.01 OR 4.29, 95% CI 1.38–13.32

During follow-up, spirometry was performed in all 58 patients. At Visit 1, the 22 patients with AAI showed significantly lower FEV1% values than patients without AAI (70.91 ± 25.60 vs 89.14 ± 17.07, p = 0.002). At visit 2 the FEV1% difference remained statistically significant (patients with AAI: 76.30 ± 24.54 vs patients without AAI: 92.91 ± 13.89, p = 0.002), whereas at visit 3 the difference was no longer statistically significant (see Table 3).

## Discussion

The aim of this study was to verify whether acute *M. pneumoniae *and *C. pneumoniae *infection is associated with the severity of acute asthma attack. We found that patients with serologically demonstrated AAI presented in the Emergency Room with a higher degree of functional impairment. However, in our case series, the role of *C. pneumoniae *was predominant, since we found only two cases of single acute *M. pneumoniae *infection. The incidence of these two bacteria observed in our study is different from previous published data [3]. This difference may be related to the long epidemic cycle of the two bacteria and to the difference of our study design that was not an epidemiological study.

The relationship between respiratory infections and asthma exacerbation has been observed since the '80s in both children and adults. First reports analysed the role of respiratory viruses, whereaslater on several observations pointed out the possible involvement of atypical bacteria in asthma, particularly *M. pneumoniae *and *C. pneumoniae *both in chronic stable asthma and in acute exacerbations [2,3,10,11,15,18-20]. Subsequently, it has been shown that *C. pneumoniae *chronic infection is associated with a more severe chronic functional impairment both in children and adults [7-9,21]. Interestingly, Wark et al. [22] showed that patients with acute asthma and an acute rise in *C. pneumoniae *antibody levels exhibit more intense airway inflammation compared to subjects with no antibody response. Recent evidence indicates that *C. pneumoniae *elements, through the activation of transcription factors (such as nuclear factor κB), are responsible for the activation of most cellular elements in bronchial tissue (epithelium, endothelium, monocytes/macrophages, smooth muscle cells), resulting in a cascade of cytokine release and adhesion molecule up regulation which favours cellular influx into the airways, persistent infection, and airway remodelling [12,23,24]. Data concerning the pathogenetic role of *M. pneumoniae *in asthma are few. It is been shown that *Mycoplasma *infection induces a secretion of IL-8 and TNF-α by human lung epithelial cells in vitro [25]. Furthermore, a potential interaction between infection and allergy is suggested by the demonstration that, in tissue biopsy in patients with asthma and positive PCR for *M. pneumoniae *and *C. pneumoniae*, there is evidence of a greater mast cell tissue infiltration than in those without infection [26].

To our knowledge, no studies have evaluated the relationship between these two atypical bacteria and the severity of functional impairment during AEBA.

Therefore, we investigated whether AAI might be associated with an acute asthma exacerbation with a particularly severe functional impairment. We found that patients with AAI exhibited on admission a more severe functional impairment. Interestingly, the majority of these presented to the Emergency Room with an acute attack classified as "severe" according to the BTS criteria, as opposed to patients without AAI. In addition, to a more severe asthma attack presentation, these patients also showed a slower rise of FEV1 during follow-up when compared with the group without AAI. A recent study showed that *C. pneumoniae *infection enhanced the proliferation and survival of immune and inflammatory cells, resulting in steroid resistance [27]. This phenomenon might explain the slower clinical improvement in these patients with AAI, where the presence of *C. pneumoniae *was predominant.

The first limitation of our study is that the diagnosis of AAI remains difficult because of the absence of well standardised diagnostic tests. However, for the diagnosis of *C. pneumoniae *infection we used the microimmunofluorescence (MIF) test that is considered the "gold standard", despite its many known limitations including subjectivity (operator dependence) [16,28]. Whilst serology is an indirect measure of infection, direct detection of *C. pneumoniae *and *M. pneumoniae *can be achieved by culture of the organisms or detection of DNA by PCR. However, culture has very poor sensitivity and is rarely successful, while PCR testing methods are not yet standardized [16,28]. In fact, we found *C. pneumoniae *PCR positivity in only around 65% of patients with serologically demonstrated acute *C. pneumonia *infection. Another limitation of our study is the small sample size, characterising a typical preliminary study. A further weakness of our work is the use of PEF expressed as a percentage of predicted value and not of the patient's previous best value, as suggested by the BTS guidelines [14]. However, in patients admitted to an Emergency Room the information about their best PEF value is not always available. In this case, as suggested by the guidelines, the percentage of predicted value could be a guide to the severity.

## Conclusion

Our data suggest an association between AAI and the severity of acute asthma attack. Considering that all patients eventually reached similar FEV1 values, we suggest that, in our study population, the diversity in exacerbation intensity may have been associated with the presence of AAI. Taking into account the limited population size in the study, our data should be confirmed by further studies. Should the association between AAI and severity of asthma exacerbation be confirmed in larger case series, appropriate anti-atypical bacterial antibiotic treatment may have to be considered in patients with severe asthma exacerbations.

## Competing interests

The authors declare that they have no competing interests.

## Authors' contributions

RC, PT, FB conceived the study. RC, PT, FB designed the trial. RC, CC, GG, AMB supervised the conduct of the trial and data collection. SA, MP, FT collected and managed the data, including quality control. RC provided statistical advice on study design and analyzed the data. RC, PT, AMB drafted the manuscript, and all authors contributed substantially to its revision. RC takes responsibility for the paper as a whole. All the authors read and approved the final manuscript.
